# Strengthening the Detection of and Early Response to Public Health Emergencies: Lessons from the West African Ebola Epidemic

**DOI:** 10.1371/journal.pmed.1001804

**Published:** 2015-03-24

**Authors:** Mark J. Siedner, Lawrence O. Gostin, Hilarie H. Cranmer, John D. Kraemer

**Affiliations:** 1 Massachusetts General Hospital Center for Global Health, Boston, Massachusetts, United States of America; 2 Harvard Medical School, Boston, Massachusetts, United States of America; 3 O’Neill Institute for National and Global Health Law, Georgetown University, Washington, D.C., United States of America; 4 Department of Health Systems Administration, Georgetown University, Washington, D.C., United States of America

## Abstract

Mark Siedner and colleagues reflect on the early response to the Ebola epidemic and lessons that can be learned for future epidemics.

**Summary Points**

- The international response to the West African Ebola virus disease epidemic has exemplified the great potential of the global public health community. However, the protracted early response also revealed critical gaps, which likely resulted in exacerbation of the epidemic.
- It is incumbent on international health partners to learn from missteps that occurred in the early stages of the epidemic and strengthen our public health capacity to better respond to future public health emergencies.
- Strategies to consider include development of a more precise system to risk stratify geographic settings susceptible to disease outbreaks, reconsideration of the 2005 International Health Regulations Criteria to allow for earlier responses to localized epidemics before they reach epidemic proportions, increasing the flexibility of the World Health Organization director general to characterize epidemics with more granularity, development of guidelines for best practices to promote partnership with local stakeholders and identify locally acceptable response strategies, and, most importantly, making good on international commitments to establish a fund for public health emergency preparedness and response.
- The recent success of the global action to stem the Ebola virus disease epidemic is laudable but should not encourage complacency in our efforts to improve the global public health infrastructure.

In March 2014, Guinea identified 49 cases of Ebola virus disease (EVD) and reported them to international health agencies [1]. Nearly twelve months later, the epidemic, having exploded into neighboring Sierra Leone and Liberia, has reached over 22,000 cases and nearly 9,000 deaths. The toll on human life, impact on health infrastructure, diversion of funding from routine—but critical—priorities, and concentrated mortality among health care workers places the epidemic among the worst disease outbreaks in recent history.

After a delayed response [2], the World Health Organization (WHO) laid out a programmatic roadmap to mobilize financial support and human resources [3]. In addition, for the first time in its history, the United Nations (UN) Security Council has authorized an emergency health mission, the UN Mission for Ebola Emergency Response, with assets typically committed to peacekeeping. The massive response that followed, involving multiple foreign governments, multinational partners, and regional ministries of health, has brought unprecedented resources to the West African region. Towards the end of 2014, the epidemic showed signs of coming under control, particularly in Guinea and Liberia. Compared to worst-case scenario estimates from earlier in the epidemic, this global response has likely saved many thousands of lives [4].

But while the international response has become an example of the great potential of the global public health community, it also revealed critical weaknesses. Had these same partners responded earlier and more effectively after the first signs of an uncharacteristic outbreak, it is likely that the number of lives lost, the impact on health infrastructure, and the magnitude of the eventual response could have been drastically diminished. It is incumbent upon the global public health community to identify gaps revealed during the early stages of the epidemic so that we improve our collective ability to detect and respond early to the inevitable next emerging disease. We offer lessons from the West African Ebola epidemic and propose solutions for future international health emergencies.

## Location, Location, Location

Experts have observed that large-scale threats from EVD are limited primarily to countries with weak public health systems [5]. The current epidemic has supported, if not confirmed, this observation. Previous EVD epidemics, almost all of which occurred in low-and-middle-income countries (LMICs) and predominantly in rural areas, have been controlled within 18 weeks, with the largest prior outbreak claiming less than 300 lives. In contrast, the current West African outbreak has now killed more people than all previous EVD outbreaks combined. Whereas WHO generally considers the health infrastructure of involved countries when assessing the risk of a potential public health emergency, this outbreak has revealed that a more granular consideration of risk will be of value. Guinea, Sierra Leone, and Liberia are all recovering from prolonged periods of civic unrest and suffering from decimated health systems with limited human resource capacity and thus demonstrate that all LMICs should not be considered the same. For example, Nigeria, another country broadly characterized as a LMIC, provides a clear illustration of how a functional, albeit limited, public health infrastructure can successfully bring an EVD outbreak under control [6]. The country responded rapidly through efforts in public education, isolation, quarantine, contact tracing, and case identification to control an epidemic after only 20 cases and 8 deaths in a little over a month.

Consequently, when a disease of epidemic potential emerges, the international community should pay increased attention to the capacity of the local health system. For example, WHO could create and maintain a curated scoring system of LMICs to include standard measures of health infrastructure, including the availability and sufficiency of the health care work force, surveillance and laboratory capacity, and personal protection equipment availability and supply chains. Moreover, a careful analysis of factors that contributed to variations in epidemic severity might lead to identification of additional characteristics to include in a ranking system. For example, local burial practices, having had prior local experience with a similar outbreak, and urban versus rural environments appear to have contributed to variations in the severity of recent West African EVD epidemics. With a more precise risk stratification system, WHO and international partners could give expedited and focused attention to countries on the list that have a particularly weak health infrastructure. This would facilitate faster and stronger responses to both routine and extraordinary health threats, as well as help to target routine support for health systems strengthening.

## Back to the Drawing Board

WHO primarily relies upon the revised 2005 International Health Regulations (IHR) to define a public health emergency of international concern (PHEIC), and to determine when such announcements should be made to alert the global community. To improve their predictive accuracy and effectiveness, WHO should reexamine the IHR’s criteria for declaring a PHEIC following each major public health emergency. This strategy will enable WHO to incorporate the lessons learned from each event to guide future responses. Multiple characteristics of the West African EVD epidemic have revealed areas for potential improvement.

First, this outbreak exemplified the importance of an often neglected criterion in the IHR—i.e., paying particular attention to cases in which “external assistance [is] needed to detect, investigate, respond, and control” the incident [7]. Although this criterion is included in the IHR as a consideration, it is subordinated to others, such as the present number of cases, which risks missing detection of threats before they reach epidemic proportions. This epidemic, in which the regional health infrastructure was quickly overwhelmed, taught us that a need for external assistance ought to become a primary condition for declaring a PHEIC. Doing so would assist struggling member states with weak health infrastructures in the crucial early stages of response.

Second, the IHR partially define a PHEIC as a disease outbreak that “constitutes a public health risk to other States through the international spread of disease,” or poses a “significant risk of international travel or trade restrictions” [7]. By this definition, outbreaks must transcend a national border before they legitimately trigger an international response. From the standpoint of national sovereignty, this requirement is understandable. States retain authority to handle health threats purely within their territories. However, the current epidemic began in December 2013, months before it crossed borders into both Sierra Leone and Liberia. This realization should challenge us to reconsider whether national borders should have such clout in considerations of potential global health emergencies. At the very least, the risk of international spread—particularly to other countries with weak public health infrastructures—should be carefully examined to prevent epidemics from growing out of control.

A more modern approach to global public health should de-emphasize the a priori criterion of international spread. Modeling studies have demonstrated that, for pathogens with relatively short generation times, early cross-border coordination between nations is crucial when epidemic diseases arise in border regions [8]. But even for diseases with relatively long generation times, as with the current EVD epidemic, waiting for cases to cross borders can result in disastrous delays. As such, removing the requirement that an outbreak be an “international threat”—or at minimum interpreting that criterion liberally—will decrease delays between reporting and response and allow much needed support to at-risk countries before epidemics have reached a tipping point.

Lastly, in hindsight this outbreak might have been defined as a public health emergency long before the WHO Director General (DG) declared it a PHEIC, and the delay probably exacerbated the slow global response to the outbreak. There are reasons not to prematurely declare a PHEIC. Doing so could damage already vulnerable political systems, lead to misallocation of scarce global health funding, and diminish the influence of the IHR. Additionally, a PHEIC declaration may inadvertently trigger harmful measures, such as border closings and travel bans, which can hinder the response to an epidemic. However, the current epidemic has taught us that delaying an announcement imperils lives and health systems and, in the long run, may do more economic harm [9], cost more response dollars, and undermine domestic political legitimacy much more than an early, errant PHEIC declaration.

Therefore, for high-consequence diseases of epidemic potential, a precautionary principle should take precedence. The WHO DG should be empowered to declare PHEICs early if criteria are met. In fact, the DG has broad discretion to determine when an outbreak constitutes a PHEIC [7]. WHO should interpret this discretion to declare graduated PHEICs, ranging from high-consequence localized events (e.g., the current Ebola outbreak as of June 2014) to clear global threats (e.g., severe acute respiratory syndrome in 2003). It can then tailor its recommendations, as well as funding needs, as the scenario unfolds. In doing so, we could expect PHEIC announcements for outbreaks that never reach devastating proportions. A similar approach has been implemented in other domains—such as genocide prevention—in which a concerted international response is required to prevent urgent problems before they become catastrophic [10]. In fact, the Emergency Response Framework, also published by WHO, which aims to grade and motivate responses to international emergencies, public health or otherwise, specifically includes a “no regrets” policy, stating that “it is better to err on the side of over-resourcing the critical functions rather than risk failure by under-resourcing” [11]. Although it garnered relatively less attention than the PHEIC announcement, WHO declared the 2014 West Africa EVD epidemic a Grade 3 International Emergency (the highest classification) on July 24, two weeks before the PHEIC was declared [12]. Similar discretion for declaring a PHEIC might help prevent the next large epidemic from gaining such momentum before the international community responds appropriately.

## Know (and Involve) Your Community

Overwhelmed and under-resourced, the affected countries have implemented drastic measures to control the epidemic, including hospital and school closures, local and national quarantines, and border closures [13]. Not surprisingly, these measures have engendered widespread public distrust of health authorities [14]. In contrast, prior successful responses to Ebola [15,16] and other epidemics [17] have prioritized early partnerships with local authorities, anthropologists, and civil society to establish buy-in from multiple stakeholders. This strategy helps ensure that the design and implementation of control measures are culturally appropriate. In hindsight, some of the negative fallout from decisions to use extraordinary measures might have been avoided had WHO, in partnership with local community leaders and public health experts, more assertively used their legitimacy to caution against the use of coercive measures without an evidence base.

When acting under emergency powers—and especially when using extraordinary control measures such as regional quarantines—governments should prioritize formation of community advisory bodies in the affected region. A rich institutional knowledge about best practices for community advisory boards exists from the research community [18,19] and, in combination with recent experience gained through collaborations with community leaders during the current epidemic, can serve as the basis for much-needed guidelines for public health activities. Members should represent divergent interests and include religious leaders, community representatives, nongovernmental organizations (NGOs), and other stakeholders. The body should be briefed on the status of the threat and called on to offer recommendations on community desensitization, capacity building, and control measures. While national and local governments hold primary responsibility for creating community advisory bodies, international actors should provide support, including technical assistance for overstretched ministries as they weigh evidence about appropriate interventions.

## Putting Money Where Our Mouths Are

Declaring a PHEIC is a critical early step to marshal an effective response. A PHEIC announcement alerts partners and should initiate a concerted, coordinated response. But a PHEIC is of little value without corresponding ammunition. For example, although the PHEIC was announced on August 6, six weeks passed between the declaration and the United States government’s commitment of US$750 million and the planned deployment of 3,000 military personnel and 65 Public Health Service commissioned corps officers [20]. In the interim, total cases of EVD increased from approximately 1,100 to over 6,500, and confirmed deaths more than tripled, from under 1,000 to approximately 3,000 [21].

The steady march of the epidemic, not only before the PHEIC declaration but also long after it, reinforces the fact that global health preparedness is contingent on the immediate availability of funding and human resources to respond. This need can be fulfilled through the establishment of a global emergency fund and the formation of a corps of trained health workers that can be deployed rapidly to curb an outbreak, with expertise ranging from epidemic surveillance to supply chain management. An International Health Systems fund, through a sustained investment by global partners, would provide much needed preparedness in future cases of outbreaks in LMICs, where local resources are not capable of controlling epidemics [22].

In 2011, a WHO committee proposed the establishment of a US$100 million contingency fund for rapid response in a declared PHEIC [23]. However, the commitment remains unfulfilled. Instead, the Organization has been forced to rely on mobilizing funding from member states and other partners [24], which inevitably delays a robust international response. Spurred by the current EVD epidemic, in January 2015 WHO’s secretariat repeated calls for the creation of a rapid response fund, to be financed by member states [25], and WHO’s executive board agreed to establish the fund [26]. Yet, the onus remains on WHO and the international community to follow through with this request even after the present emergency has faded and ensure this call to action does not meet a similar fate as previous attempts.

## There Is No Substitute for Prevention

Arguably the greatest lesson to emerge from the Ebola virus epidemic is that both national ministries and the global public health community were caught off guard and unprepared [27]. In addition to the many thousands of lives taken from both Ebola infections and interrupted access to routine health services, billions of dollars that could have otherwise been used for development and health systems strengthening were allocated to direct the Ebola response. The deleterious impact on local economies is equally staggering. The World Bank predicts that the three most affected countries will lose US$1.6 billion in economic growth in 2015, corresponding to an average gross domestic product (GDP) loss of 12% across the three countries. In Sierra Leone, a loss of nearly 20% of the GDP is projected [28]. All this resulted from a disease that is relatively easy to control in settings with established health systems. Had a disease like SARS, with airborne transmission and a high case-fatality rate emerged in a similar location, the fallout could have been far worse.

What can be done today to prepare for the unavoidable public health threats of tomorrow? In the long term, it will be essential to build more robust health systems. Beyond public health emergencies, strong health systems will improve the health and wellbeing of the population in LMICs by delivering a range of essential services [29–31]. The IHR currently mandate that technical support—surveillance, epidemiology, and laboratory and other core capacities—be provided by high-income countries to low-income countries to build capacity to survey and respond to outbreaks and that a detailed international framework exist for defining and assessing these capacities [32]. However, although this is a central component of the obligations borne by countries for collective health security, capacity building support to low-income countries has long been underfunded. As a result, most countries in sub-Saharan Africa continue to suffer from weak systems for disaster preparedness and emergency response [32,33].

To ensure a truly robust response to global health hazards, states must abide by their IHR obligations to build core public health capabilities in regions of need. Human resources capacity building, laboratory infrastructure, and epidemiologic surveillance expertise are all urgently needed in West Africa and beyond. These inputs should be routinely assessed through a demonstrated capability to deploy resources successfully and in a timely fashion to respond to emergencies [34]. Moreover, such investments clearly will have secondary benefits for routine health services in the areas they are employed. While successful implementation of these elements of the IHR will require substantial resource expenditures by high-resource countries, it is a legal and moral duty to which wealthy countries bound themselves when joining the IHR. The EVD epidemic has brought broad realization that health systems strengthening will be crucial to realize the benefits of a global community protected against international infectious disease threats. A positive legacy of the otherwise disastrous EVD outbreak should be a global community with renewed commitment to the establishment of a capable emergency response infrastructure.

## Conclusions

While current efforts to bring the EVD epidemic under control should be widely applauded, the delayed response during the early stages of the EVD epidemic in West Africa exemplifies not only the danger posed by disease outbreaks in states with weak health systems but also their widespread impact in an increasingly globalized world. The international public health community—WHO, states, and stakeholders—can learn from missteps during the first stages of the epidemic. If instead we accept the status quo by relying on overwhelmed and undersupported domestic health systems and international charity to respond to threats after they have become emergencies, history will repeat itself. At stake are the values of the IHR and the legitimacy of WHO. The power of global health law and global health institutions will remain seriously unrealized and deeply compromised if the Ebola epidemic does not spur fundamental reform.

## Abbreviations

DG: director general
EVD: Ebola virus disease
GDP: gross domestic product
IHR: International Health Regulations
LMICs: low-and-middle-income countries
NGO: nongovernmental organization
PHEIC: public health emergency of international concern
UN: United Nations
WHO: World Health Organization
